# Turbulence Model Comparative Study for Complex Phenomena in Supersonic Steam Ejectors with Double Choking Mode

**DOI:** 10.3390/e24091215

**Published:** 2022-08-30

**Authors:** Yiqiao Li, Chao Niu, Shengqiang Shen, Xingsen Mu, Liuyang Zhang

**Affiliations:** National Joint Engineering Research Center for Thermal Energy Integration, School of Energy and Power Engineering, Dalian University of Technology, Dalian 116024, China

**Keywords:** steam ejector, turbulence models, non-equilibrium condensation, shock wave, boundary layer separation

## Abstract

Scholars usually ignore the non-equilibrium condensing effects in turbulence-model comparative studies on supersonic steam ejectors. In this study, a non-equilibrium condensation model considering real physical properties was coupled respectively with seven turbulence models. They are the *k-ε* Standard, *k-ε* RNG, *k-ε* Realizable, *k-ω* Standard, *k-ω* SST, Transition SST, and Linear Reynolds Stress Model. Simulation results were compared with the experiment results globally and locally. The complex flow phenomena in the steam ejector captured by different models, including shock waves, choking, non-equilibrium condensation, boundary layer separation, and vortices were discussed. The reasons for the differences in simulation results were explained and compared. The relationship between ejector performance and local flow phenomena was illustrated. The novelty lies in the conclusions that consider the non-equilibrium condensing effects. Results show that the number and type of shock waves predicted by different turbulence models are different. Non-equilibrium condensation and boundary layer separation regions obtained by various turbulence models are different. Comparing the ejector performance and the complex flow phenomena with the experimental results, the *k-ω* SST model is proposed to simulate supersonic steam ejectors.

## 1. Introduction

Steam ejectors are widespread in refrigeration [1,2], desalination [3,4], etc. They are energy-saving and environmentally friendly fluid machines, which can use the residual pressure of steam to recover low-pressure steam without additional consumption of mechanical energy [5].

There are complex supersonic flow phenomena in a steam ejector, such as shock waves [6], choking [7,8], non-equilibrium condensation [9], boundary-layer separation, and vortices [10]. It is hard to rely on experiments to investigate the internal process thoroughly, especially to achieve accurate and interference-free measurements in the supersonic fluid because of the flow field involving high velocity, large gradients of the fluid properties, and rapid phase transition. Computational fluid dynamics is an effective way to achieve specific analysis in the micro process and provide a basis for studying the physical essence.

RANS (Reynolds Average Navier-Stokes) is the most widely used computational method in the study of the complex flow phenomena in steam ejectors. Wang et al. [7] adopted the *k-ε* Realizable turbulence model to simulate primary pseudo-shock patterns and choking flow in a steam ejector. They stated that when the primary pseudo-shock overexpands, the over-choking condition occurs. Kong [11] adopted the *k-ω* SST model and pointed out that vortices are formed by the combined action of boundary layers and shock waves in a supersonic ejector. Bartosiewicz et al. [6] adopted the *k-ε* Standard turbulence model to investigate the relationship between shock waves, boundary-layer separation, and ejector efficiency. They pointed out that separation intensity increases with increasing shock wave intensity, and ejector efficiency decreases with increasing separation intensity. 

No agreement has been reached yet on the closures for the turbulence model in the Reynolds Averaged Navier_º–_Stokes (RANS) approach to simulate a supersonic steam ejector [12,13]. Turbulence models of interest to scholars include the *k-ε* Standard, *k-ε* RNG, *k-ε* Realizable, *k-ω* Standard, *k-ω* SST, Transition SST, and RSM. Ruangtrakoon et al. [14] neglected condensing effects and compared the *k-ε* Realizable and *k-ω* SST. They concluded that simulation results of the *k-ω* SST agree best with the experimental results of the ejector performance (*Er* and *P*_d_*). Besagni et al. [13] ignored the condensing effect and compared seven turbulence models (*k-ε* Standard, *k-ε* RNG*, k-ε* Realizable *k-ω* Standard, *k-ω* SST, Spalart-Allmaras, and RSM). They found that *Er* and the static wall pressure profiles predicted by *k-ω* SST are in best agreement with the experiment. Han [10] neglected condensing effects and compared the static wall pressure profiles of a steam ejector measured by the experiment with those predicted by four turbulence models (*k-ε* Standard, *k-ε* RNG*, k-ε* Realizable, and *k-ω* SST). The results showed that the minimum relative error is obtained by the *k-ε* Realizable model. Varga et al. [15] neglected condensing effects and evaluated six turbulence models (*k-ε* Standard, *k-ε* RNG*, k-ε* Realizable, *k-ω* Standard, *k-ω* SST, and Transition SST). They indicated that the Transition SST accord best with the experimental results of *Er* and *P*_d_*.

Scholars usually ignore the non-equilibrium condensing effects in the turbulence-model comparative studies on supersonic steam ejectors. However, non-equilibrium condensation surely causes significant influences on the ejector performance and flow characteristics [16]. Sharifi et al. [17] reported that the condensation occurring in the supersonic area leads to the Ma reduction and ejector performance increase. Wang et al. [18] found that condensation leads to an increase in the critical discharge pressure, and the ideal gas model cannot take the influence into account. Ariafar et al. [19] stated that compared with the ideal-gas model, their condensation model gets a 10% higher *Er* and 7% higher *P*_d_*. Yang et al. [20] pointed out that, compared to the condensation model, the dry-gas model gets lower temperature and higher *Er*. Consequently, the comparative study of non-equilibrium condensation turbulence models for complex flow phenomena in supersonic steam ejectors is urgently needed.

In this study, a non-equilibrium condensation model considering real physical properties was coupled with seven turbulence models, respectively. The turbulence models are the *k-ε* Standard, *k-ε* RNG, *k-ε* Realizable, *k-ω* Standard, *k-ω* SST, Transition SST, and Linear RSM. Simulation results were compared with the experiment results globally and locally. The complex flow phenomena in the steam ejector captured by different models, including shock waves, choking, non-equilibrium condensation, boundary layer separation, and vortices were discussed. The reasons for the differences in simulation results were explained and compared. The relationship between ejector performance and local flow phenomena was illustrated. This paper’s novelty lies in the conclusions that consider the non-equilibrium condensing effects. These findings can provide guidance and a reasonable basis for the selection of turbulence models in supersonic steam ejectors.

## 2. Numerical Simulation Method

### 2.1. Mathematical Model

Fundamental equations governing behaviors of the wet steam at a steady state in the supersonic steam are the three conservation equations, which are the mass, momentum, and energy-conservation equations:

(1) $$\frac{\partial }{\partial x_{j}}\left(\rho v_{j}\right)=0$$

(2) $$\frac{\partial }{\partial x_{j}}\left(\rho v_{j}v_{i}\right)=\frac{\partial \tau_{ij}}{\partial x_{j}}-\frac{\partial P}{\partial x_{i}}$$

(3) $$\frac{\partial }{\partial x_{j}}\left[v_{j}\left(\rho E+P\right)\right]=\frac{\partial }{\partial x_{j}}\left(\lambda_{eff}\frac{\partial T}{\partial x_{j}}\right)+\frac{\partial }{\partial x_{j}}{\left(v_{i}\tau_{ij}\right)}_{\;}$$

where

(4) $$E=h-\frac{p}{\rho }+\frac{1}{2}v_{j}v_{j}$$

The seven turbulence models are for the compressible N-S equations, respectively.

The third-order virial-type equation [21] is used to express the real state of steam:

(5) $$P=\rho_{v}RT\left(1+B\rho_{v}+C\rho_{v}{}^{2}\right)$$

where:

(6) $$B=a_{1}{\left(1+\frac{\tau }{\alpha }\right)}^{-1}+a_{2}e^{\tau }{\left(1-e^{-\tau }\right)}^{\frac{5}{2}}\tau^{-\frac{1}{2}}+a_{3}\tau$$

where *τ* = 1500/*T*, *α* = 10000.0, *α*_1_ = 0.0015, *α*_2_ = −0.000942, and *α*_3_ = −0.0004882.

(7) $$C=a\left(\tau {}^{\prime }-\tau_{0}\right)e^{-\alpha \tau {}^{\prime }}+b$$

where *τ^′^* = *T*/647.286, *τ*_0_ = 0.8978, *α* = 11.16, *a* = 1.772, and *b* = 1.5 × 10^−6^

The vapor properties [22] are:

(8) $$Cp_{v}=Cp_{0}\left(T\right)+R\left[\left[\left(1-\alpha_{v}T\right)\left(B-B_{1}\right)-B_{2}\right]\rho_{v}+\left[\left(1-2\alpha_{v}T\right)C+\alpha_{v}TC_{1}-\frac{C_{2}}{2}\right]\rho_{{}_{v}}{}^{2}\right]$$

(9) $$Cv_{v}=Cp_{0}\left(T\right)-R\left[1+\left(2B_{1}+B_{2}\right)\rho_{v}+\left(C_{1}+C_{2}/2\right)\rho_{{}_{v}}{}^{2}\right]$$

where *Cp*_0_ is an isobaric specific heat when *P* is equal to 0:

(10) $$C_{P0}\left(T\right)=\sum_{i=1}{}^{6}a_{i}T^{i-2}$$

where *α*_1_ = 46.0, *α*_2_ = 1.47276, *α*_3_ = 8.38930 × 10^−4^, *α*_4_ = −2.19989 × 10^−7^, *α*_5_ = 2.46619 × 10^−10^, and *α*_6_ = −9.70466 × 10^−14^. $B_{1}=T\frac{dB}{dT},C_{1}=T\frac{dC}{dT},B_{2}=T^{2}\frac{dB^{2}}{dT^{2}}$, and $C_{2}=T^{2}\frac{dC^{2}}{dT^{2}}$

(11) $$h_{v}=h_{0}\left(T\right)+RT\left[\left(B-B_{1}\right)\rho_{{}_{v}}+\left(C-\frac{C_{1}}{2}\right)\rho_{v}{}^{2}\right]$$

(12) $$s_{v}=s_{0}\left(T\right)-R\left[\ln\rho_{v}+\left(B+B_{1}\right)\rho_{v}+\left(\frac{C+C_{1}}{2}\right)\rho_{{}_{v}}{}^{2}\right]$$

where

(13) $$h_{0}\left(T\right)=\int C_{P0}dT+h_{c}$$

(14) $$s_{0}\left(T\right)=\int C_{P0}dT+s_{c}$$

where *h*_c_ and *s*_c_ are arbitrary constants equal to 1811.06 kJ/kg and 0.97012 kJ/kg, respectively.

(15) $$\mu_{v}=\left(-15.371+99.871\tau {}^{\prime }-133.993\tau {}^{\prime }{}^{2}+75.8226\tau {}^{\prime }{}^{3}\right)\times 10^{-6}$$

The thermal conductivity *λ*_v_ is the piecewise function of *T*, which refers to the reference [22] 

The equation for the saturated vapor line [21] is:

(16) $$\frac{P_{\mathrm{sat}}}{P_{c}}=\exp\left[0.01\frac{T}{\tau {}^{\prime }\left(T-1)\right)}\sum_{i=1}^{8}a_{i}{\left(3.3815-T\right)}^{i-1}\right]$$

where *P*_c_ = 220.98 bar, *a*_1_ = −741.9242, *a*_2_ = −29.721, *a*_3_ = −11.55286, *a*_4_ = −0.8685635, *a*_5_ = −0.1094098, *a*_6_ = 0.439993, *a*_7_ = 0.2520658, *a*_8_ = 0.05218684.

The equation for the saturated liquid line [21] is:

(17) $$\rho_{l}=\sum_{i=0}^{3}a_{i}\tau {}^{\prime }$$

where *a*_0_ = 928.08, *a*_1_ = 464.63, *a*_2_ = −568.46, *a*_3_ = −255.17.

The liquid properties [23] are given by:

(18) $$\sigma =0.08227+0.075612\tau {}^{\prime }-0.256889\tau {}^{\prime }{}^{2}+0.095928\tau {}^{\prime }{}^{3}$$

(19) $$Cp_{l}=\sum_{i=0}^{5}a_{i}T$$

where *a*_0_ = −36571.6, *a*_1_ = 555.217, *a*_2_ = −2.96724, *a*_3_ = 0.00778551, *a*_4_=−1.00561 × 10^−5^, *a*_5_ = 5.14336 × 10^−9^.

(20) $$\lambda_{l}=\sum_{i=0}^{5}a_{i}T$$

where *a*_0_ = −1.17633, *a*_1_ = 0.00791645, *a*_2_ = −1.48603 × 10^−5^, *a*_3_=1.31689 × 10^−7^, *a*_4_ = −2.47590 × 10^−10^, *a*_5_=1.55638 × 10^−1^

(21) $$\mu_{l}=\sum_{i=0}^{6}a_{i}T$$

where *a*_0_ = 0.530784, *a*_1_ = −0.00729561, *a*_2_ = 4.16604 × 10^−5^, *a*_3_ = −1.26258 × 10^−7^, *a*_4_ = 2.13969 × 10^−10^, *a*_5_ = −1.92145 × 10^−13^, *a*_6_ = 7.14092 × 10^−17^.

The specific enthalpy and entropy of saturated liquid [21] are:

(22) $$h_{\mathrm{lsat}}=h_{\mathrm{vsat}}-\left(\frac{1}{\rho_{{}_{\mathrm{lsat}}}}-\frac{1}{\rho_{\mathrm{vsat}}}\right)T_{\mathrm{sat}}\frac{dP_{\mathrm{sat}}}{dT_{\mathrm{sat}}}$$

(23) $$s_{\mathrm{lsat}}=s_{\mathrm{vsat}}-\frac{h_{\mathrm{lsat}}-h_{\mathrm{vsat}}}{T_{\mathrm{sat}}}$$

Assume that *T*_l_ = *T*_v_, *P*_l_ = *P*_v_, *v*_l_ = *v*_g_. Furthermore, since the droplet diameter is very small and *ρ*_l_ is much greater than *ρ*_v_, it is approximated that:

(24) $$\rho =\rho_{v}/\left(1-\beta \right)$$

The mixture properties are derived by the following mixing law:

(25) $$\varphi_{m}=\varphi_{l}\beta +\left(1-\beta \right)\psi_{v}$$

where *φ* represents the thermodynamic properties: *h*, *s*, *Cp*, *Cv*, *μ*, or *k*, respectively, and the specific heat capacities ratio and sonic velocity of the mixture are, respectively:

(26) $$\gamma_{m}=\frac{1}{\beta P}\frac{Cp}{Cv}$$

(27) $$c_{m}=\sqrt{\gamma_{m}\frac{P}{\rho }}$$

The non-equilibrium condensation model including nucleation model and droplet growth model is coupled with the flow model. Following are the mass-fraction transport equation of the liquid-phase [24] and the density transport equation of the droplet number [25], respectively:

(28) $$\nabla \cdot \left(\rho \vec{v}\beta \right)=\Gamma$$

(29) $$\nabla \cdot \left(\rho \vec{v}\eta \right)=\rho J$$

where *Γ* is liquid mass generation rate; *J* is nucleation rate. Their equations are as follows:

(30) $$\Gamma ={\dot{m}}_{l}=-{\dot{m}}_{v}=\frac{4}{3}\pi \rho_{l}Jr_{*}{}^{3}+4\pi \rho_{l}\eta {\bar{r}}^{2}\frac{\partial \bar{r}}{\partial t}$$

(31) $$J=\frac{q_{c}}{\left(1+\theta \right)}\left(\frac{\rho_{g}^{2}}{\rho_{l}}\right)\sqrt{\frac{2\sigma }{M_{m}^{3}\pi }}e^{-\left(\frac{4\pi r_{*}^{2}\sigma }{3k_{B}T_{g}}\right)}$$

where *q*_c_ is the evaporation coefficient, *k_B_* is the Boltzmann constant, *M_m_* is the mass of a water vapor molecule, and *σ* is the surface tension of the liquid mass. r_*_, *η*, *θ* are the critical droplet radius, droplets per unit volume number, non-isothermal correction coefficient, respectively:

(32) $$r_{*}=\frac{2\sigma }{\rho_{l}RT\ln S}$$

where *S* = *P*/*P*sat.

(33) $$\theta =\frac{2\left(\gamma -1\right)}{\gamma +1}\left(\frac{h_{\mathrm{lv}}}{RT}\right)\left(\frac{h_{\mathrm{lv}}}{RT}-0.5\right)$$

(34) $$\eta =\frac{\beta }{\left(1-\beta \right)V_{d}\left(\rho_{l}/\rho_{g}\right)}$$

where *V*_d_ is the mean droplet volume:

(35) $$V_{d}=\frac{4}{3}\pi {\bar{r}}^{3}$$

Through combining *β* and *η*, the average droplet radius is:

(36) $$\bar{r}=\sqrt[{3}]{\frac{3\beta }{4\pi \eta (1-\beta )(\rho_{l}-\rho_{g})}}$$

The droplet’s growth rate equation [26] is as follows:

(37) $$\frac{\partial r}{\partial t}=\frac{P}{h_{\mathrm{lv}}\rho_{l}\sqrt{2\pi RT}}\frac{\gamma +1}{2\gamma }C_{p}\left(T_{\mathrm{sat}}-T\right)$$

where *h*_lv_ is the specific latent enthalpy that is obtained by differentiating the saturated vapor equation and using the Clausius-Clapeyron relation.

### 2.2. Numerical Scheme

The finite volume method was used to solve the 2-D axisymmetric steady-state N-S equations with seven turbulent models that are the *k-ε* Standard, *k-ε* RNG, *k-ε* Realizable, *k-ω* Standard, *k-ω* SST, Transition SST, and Linear RSM, respectively. The *k-ω* Standard model has good calculation accuracy for both near-wall and boundary-layer flow calculations. Its improved model *k-ω* SST model is more suitable for the simulation of adverse pressure gradient flow, airfoil, and transonic shock wave because it considers the transport of turbulent shear stress in the definition of turbulent viscosity. The Transition SST model is only applicable to wall-bounded flows, which is based on the coupling of the *k-ω* SST transport equation and the other two transport equations. The *k-ε* Standard model is often used in the numerical simulation of heat exchange. Its improved models, the *k-ε* RNG and *k-ε* Realizable model can handle flows with high strain rates, large streamline curvatures, vortices, and rotations better than the *k-ε* Standard model. RSM has great potential to give accurate predictions for complex flows because it accounts for the anisotropy of eddy viscosity [23].

The non-equilibrium condensation model was coupled with the flow model. The implicit solver was based on the density coupling. The Green-Gauss node-based method and the second-order upwind schemes were employed to discretize the variable gradient and the convection and diffusion terms, respectively.

When all the three criteria are met, the computation is considered to be convergent: (1) every residual term’s convergence absolute criteria is less than 10^−6^ all the time; (2) the global net flux difference/*G*_s_ is less than 10^−7^ all the time; (3) *G* is constant at the inlets and outlet.

### 2.3. Boundary Conditions

**Figure 1:**
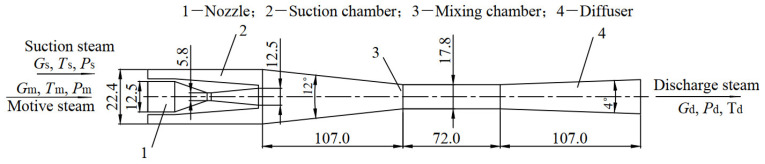
Structure and size of the steam ejector.

The ejector dimensions and computation parameters from Chen and Sun’s experiment [1] on a steam-ejector refrigeration system are illustrated in Figure 1 and Table 1, respectively. The calculated and the experimental results are compared. The inlet total pressures, outlet total pressures, and inlet total temperatures are prescribed. It is assumed that all walls are adiabatic smooth solid, and the boundary conditions are no-slip and no-penetration. Turbulent viscosity ratios of the suction and motive steam are 100 and 500, respectively. Turbulence intensity of the suction and motive steam are 2% and 5%, respectively, as proposed in the reference [13].

The strategy to achieve the solution convergence is as follows: the convergence results without the condensation model are obtained first. Then, the results are used as the initial flow field, and the condensation model is added to obtain the final simulation results.

Among the computation parameters, *P*_d_ = 1335 Pa is selected to illustrate the complex flow phenomena captured by the seven turbulence models in Chapter 3 because the double-choking characteristic is most pronounced at low discharge pressure. 

### 2.4. Meshing Approach

**Figure 2:**
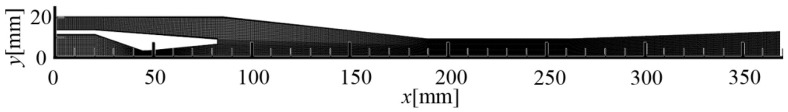
Structural diagram of the two-dimensional axisymmetric mesh.

**Figure 3:**
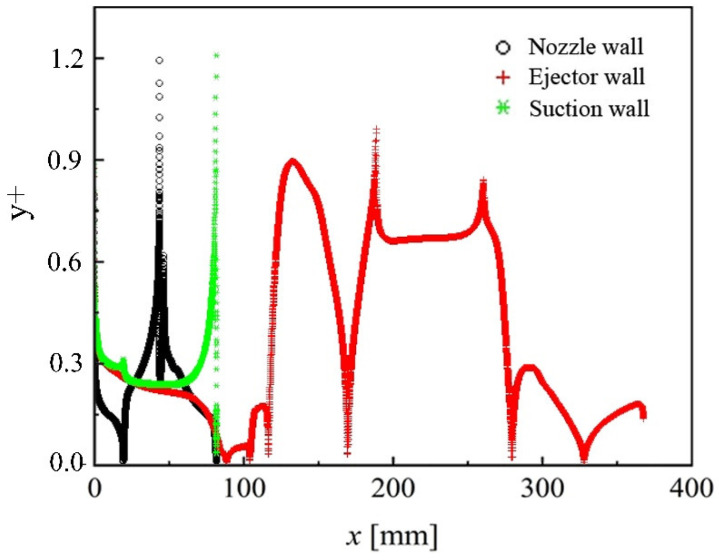
y^+^ profile along ejector wall.

A 2-D axisymmetric ejector mesh with refinement in the area of boundary layers and shock waves was established, as shown in Figure 2. The value of y^+^_max_ is less than 1.2, as shown in Figure 3. It is because when y^+^ ≈ 1, the low-Re boundary conditions that are suitable for the complex flow are adopted in the *k-ω* Standard, *k-ω* SST, and Transition SST models. Moreover, the scalable wall functions are adopted in the Linear RSM and *k-ε* series models [23]. The scalable wall functions avoid the deterioration of standard wall functions under grid refinement below y^+^ < 11 because it forces the usage of the log law in conjunction with the standard wall functions approach [23]. 

**Figure 4:**
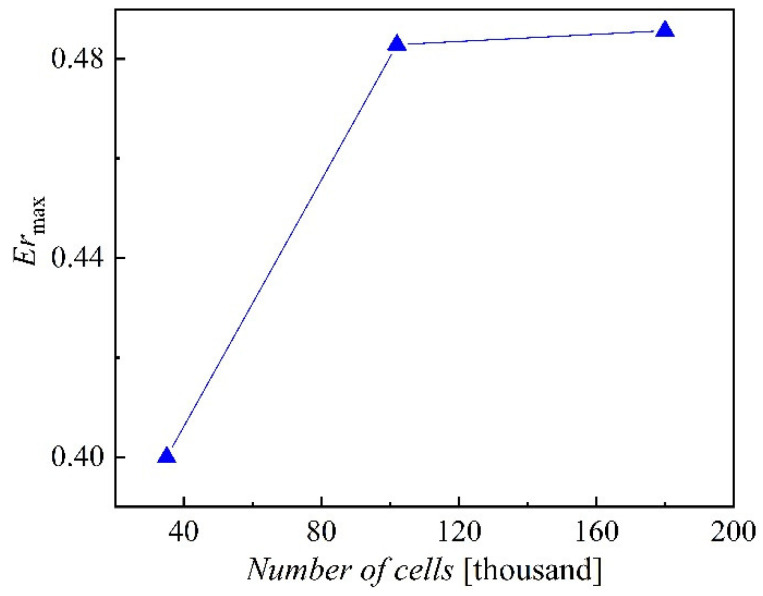
Mesh independence study.

The mesh independence study is achieved with a coarse mesh with 40,000 elements, a medium mesh with 87,000 elements, and a fine mesh with 186,000 elements. Figure 4 illustrates that the simulation results are very close for the last two meshes. The GCI method is a way to measure the relative discrete error of the computed solution with different mesh levels [27]. GCI study results indicate that the medium mesh with 87,000 elements has a low GCI value and relative discrete error, as shown in Table 2. Additionally, its solution duration and memory usage are lower than those of the fine mesh. As a result, the medium mesh with 87,000 elements is adopted in this work.

## 3. Results and Discussion

### 3.1. Turbulence Model Comparative Study for Ejector Performance

The critical discharge pressure (*P*_d_*) is the maximum discharge pressure to keep the entrainment ratio stable with a certain motive steam pressure and the suction steam pressure. The maximum entrainment ratio (*Er*_max_) is the entrainment ratio when the ejector is in a double-choking mode, whose value is equal to the ratio of the suction steam mass flow rate (*G*_s_) to the motive steam mass flow rate (*G*_m_). *P*_d_* cannot be directly available from a single simulation run. It is necessary to gradually increase the discharge pressure (preferably in increments of 0.5 kPa or less) and repeat the numerical simulation until the entrainment ratio just starts to decrease. The discharge pressure at this time is *P*_d_*.

**Figure 5:**
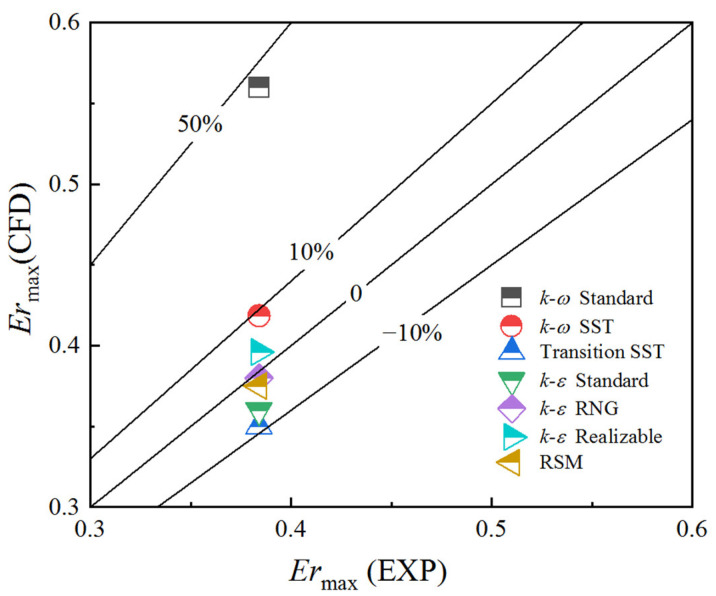
The relative error of the maximum entrainment ratio of seven turbulence models.

The *Er*_max_ obtained by the *k-ω* Standard is considerably higher than experimental data with a relative error of 46%. The *Er*_max_ predicted by the *k-ω* SST, *k-ε* Realizable, *k-ε* RNG, Linear RSM, *k-ε* Standard, and Transition SST model are within an acceptable range, as shown in Figure 5. The *k-ε* RNG has an additional term in its turbulent dissipation rate equation that improves the accuracy for rapidly strained flows. The *k-ε* Realizable contains an alternative formulation for the turbulent viscosity, and a modified transport equation for the dissipation rate has been derived from an exact equation for the transport of the mean-square vorticity fluctuation [23]. Therefore, the simulation results of the *k-ε* RNG and *k-ε* Realizable are better than that of the *k-ε* Standard.

**Figure 6:**
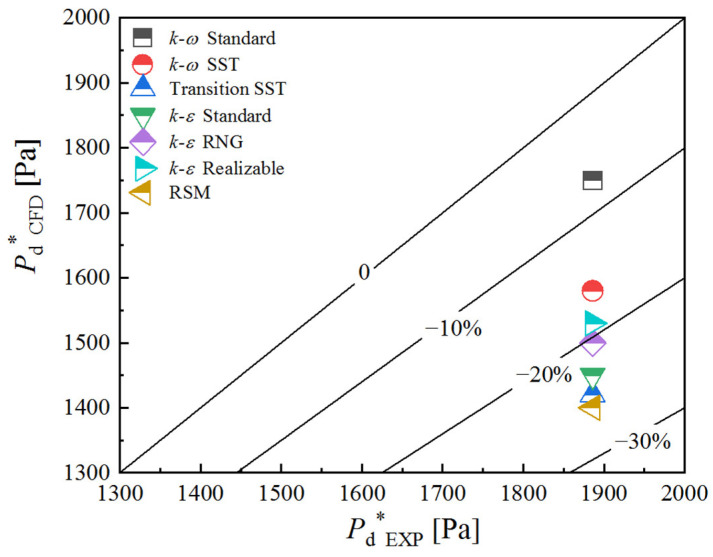
The relative error of the critical discharge pressure of seven turbulence models.

Figure 6 illustrates the relative error of *P*_d_* of the different turbulence models. The *P*_d_* predicted by the seven models are all below the experimental data, which is consistent with the findings of the majority of scholars [9,19,28,29,30,31]. The relative errors of the *k-ω* Standard, *k-ω* SST, and *k-ε* Realizable model which are all below 20% are acceptable. 

In summary, the *k-ω* SST and *k-ε* Realizable model agree with the experimental results in terms of the ejector performance.

### 3.2. Turbulence Model Comparative Study for Double Choking and Shock Waves

Steam ejectors with double choking can keep compression performance towering and stable [32]. On the one hand, shock waves in the diffuser are necessary for double choking. On the other hand, shock waves cause energy dissipation. 

**Figure 7:**
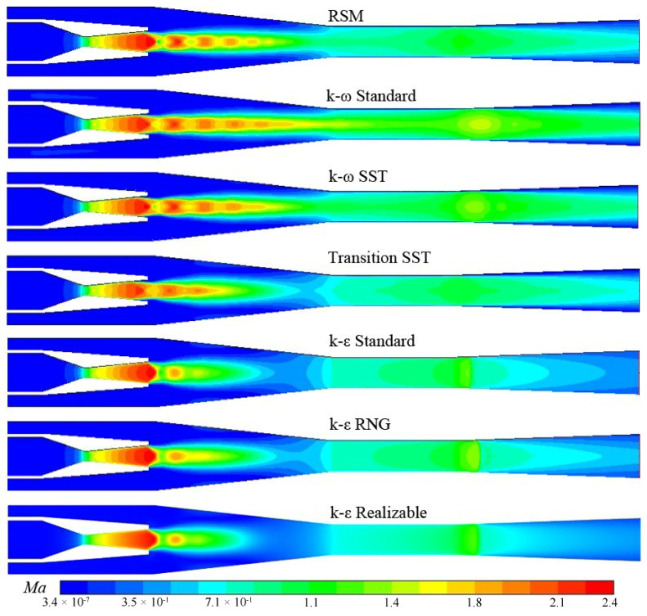
*Ma* counters with seven turbulence models.

A sudden drop of *Ma* indicates that a shockwave appears in the flow field. A sudden rise of *Ma* indicates that an expansion wave occurs. The flow direction is vertical to the surface of a normal shock wave, which does not change after the normal shock wave. The direction changes after passing through an oblique one. The number and type of shockwaves predicted by different turbulence models are different, as shown in Figure 7.

In the mixing chamber, the supersonic motive steam flowing out of the Laval nozzle is compressed because the nozzle-outlet pressure is less than the suction pressure, resulting in the formation of an oblique shock wave system intersecting at the center. The oblique shock wave passes through the axis of the ejector and is reflected as an expansion wave after reaching the mixed shear layer on the opposite side. Then the expansion wave passes through the axis of the ejector and is reflected as an oblique shock wave after reaching the shear layer located on the opposite side. After continuous reflection and conversion, the wave gets weaker and weaker until the viscous dissipation and mixing effect consume its energy over. An oblique shock wave and an expansion wave constitute a diamond shock wave. Figure 7 describes how the flow velocity predicted by *k-ω* Standard commences being supersonic from the nozzle throat and passes through the vicinity of the mixing chamber axis until multiple oblique shocks occur in the diffuser. Then the flow velocity decreases to subsonic speed. The supersonic flow around the axis predicted by the other models only appears in the diffuser and a part of the mixing chamber. 

In double-choking mode, the pressure of mixed wet steam in the diffuser must be reduced to adapt to the low discharge pressure. Therefore, shock waves appear again to consume excess energy. The *k-ε* Realizable, *k-ε* RNG and *k-ε* Standard predict a normal shock that makes the fluid speed slow sharply to subsonic. Whereas the other four models obtain oblique shock waves that make fluid velocity decrease from supersonic to subsonic gradually. Chen and Sun obtained the holographic interferogram of an air ejector and pointed out that it also can be a good indication of the flow field of a steam ejector [1]. They captured oblique shock waves in the diffuser when the ejector is in the double-choking mode. Therefore, the shock wave type in the diffuser predicted by the *k-ω* Standard, *k-ω* SST, Linear RSM, and Transition SST model is consistent with the experiment.

### 3.3. Turbulence Model Comparative Study for Condensation Phenomenon

The rapid expansion of the steam in the supersonic steam ejector leads to a deviation from the saturated equilibrium state and the formation of the subcooling flow. When the thermodynamic non-equilibrium develops to a certain limit (i.e., when the Wilson point of steam is reached), the vapor molecules will collide and adhere spontaneously to form condensed cores under the action of chemical potential. The steam condenses rapidly when the condensed cores exceed the critical scale ones, which is the non-equilibrium condensation. The two-phase mixture of saturated steam and small droplets is formed in the flow field.

**Figure 8:**
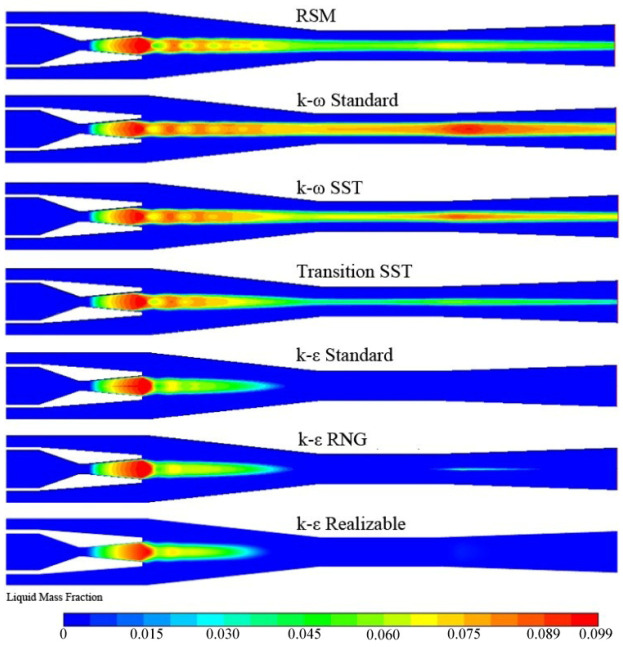
Counters of liquid mass fraction with seven turbulence models.

Figure 8 describes the condensation around the axis of mixing chamber and diffuser predicted by the Linear RSM, *k-ω* Standard, *k-ω* SST, Transition SST, and *k-ε* RNG model. The first four models predict that the condensation appears from the nozzle expansion section and passes through the vicinity of the ejector axis to the diffuser outlet. The *k-ε* RNG only predicts the condensation in the contraction of the mixing chamber and the diffuser entrance. The other two models only predict the condensation in the contraction of the mixing chamber. Chen and Sun obtained water droplets in the whole mixing chamber in visualization experiments [1]. Therefore, the simulation results of the *k-ω* Standard, *k-ω* SST, Linear RSM, and Transition SST models are consistent with the experimental results in relevant aspects.

### 3.4. Turbulence Model Comparative Study for Boundary Layer Separation and Vortex

**Figure 9:**
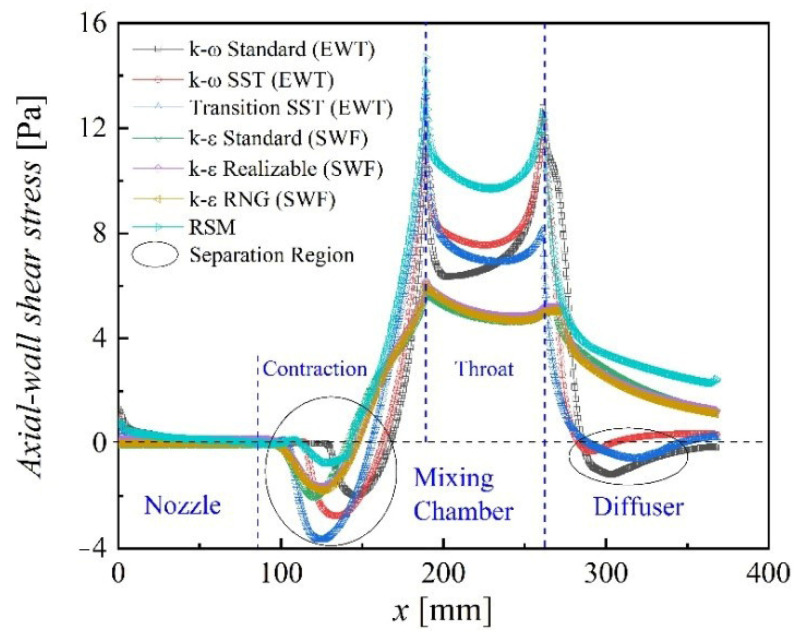
Profiles of the axial wall shear stress with seven turbulence models.

The boundary layer begins to separate when axial wall shear stress decreases by 0, as shown in Figure 9. When the axial wall shear stress rises back to 0, the separation ends. The two points are called the “separation point” and the “reattachment point”, respectively. The region between the two points is the separation region [10]. In the boundary-layer separation area, the greater the absolute value of the axial wall shear stress, the more intense the separation.

Chen and Sun found that a circulating flow exists in the edge region of the contraction of the mixing chamber in their visualization experiments [1], and the same is true in the seven turbulence models predicted. Moreover, the *k-ω* Standard, *k-ω* SST, and Transition SST also predict the boundary-layer separation in the diffuser, whereas the other models only predict that in the contraction of the mixing chamber, as shown in Figure 9. 

In the mixing chamber, the separation intensity predicted by the Transitional SST model and the Linear RSM are the highest and the lowest, respectively. The separation point obtained by the *k-ε* Standard occurs furthest upstream, i.e., closest to the inlet of the suction chamber. The separation point obtained by the *k-ω* Standard occurs farthest from the suction-chamber inlet. 

In the diffuser, the boundary-layer separation predicted by the *k-ω* SST both occurs and disappears furthest upstream. The separation intensity is the lowest. It has the smallest boundary-layer separation region. The separation point predicted by the *k-ω* Standard occurs furthest downstream. The separation intensity is the highest. The boundary layer is always in a separated state until the ejector outlet. It has the largest boundary layer separation region. The simulation results of the Transitional SST are between the above two models, and the reattachment point appears. The *k-ε* Standard, *k-ε* RNG, *k-ε* Realizable, and Linear RSM do not predict the boundary-layer separation in the diffuser because of the disadvantage that the *k-ε* series models are not sensitive to adverse pressure gradients and boundary layer separation [13].

**Figure 10:**
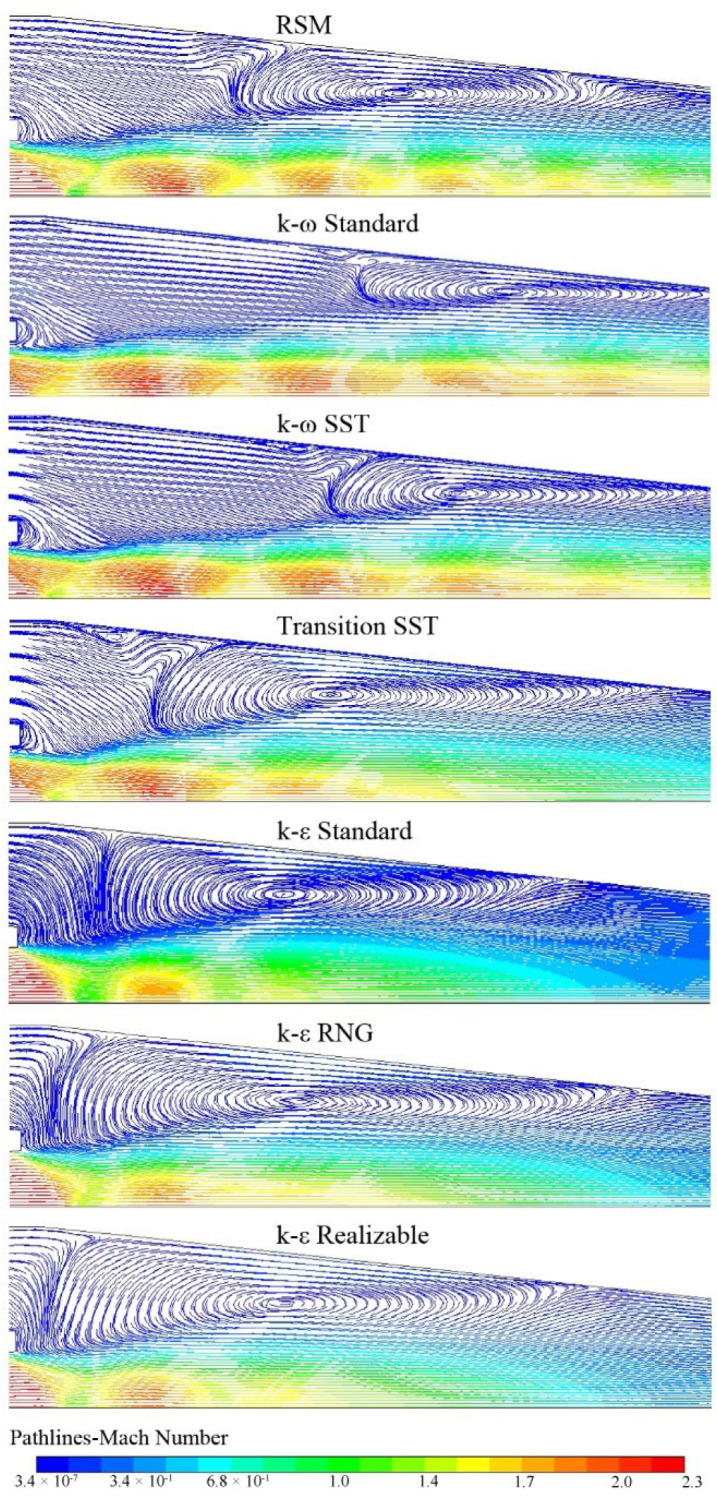
*Ma* pathlines with seven turbulence models.

Figure 10 illustrates that in the mixing chamber of the ejector, two vortices are obtained by *k-ω* Standard, *k-ω* SST, and Transition SST: a main vortex and another small vortex closer to the suction-chamber entrance, whereas only one vortex is predicted by the other models. The more intense the vortex is, the greater the energy dissipation and the lower *Er*_max_ is. The vortex areas in the mixing chamber predicted by the seven models from small to large are as follows: the *k-ω* Standard, *k-ω* SST, Linear RSM, *k-ε* Realizable, *k-ε* RNG, *k-ε* Standard, Transition SST. The *Er*_max_ predicted by different turbulence models in Figure 5 is in the same order from large to small.

## 4. Conclusions

Scholars usually ignore the non-equilibrium condensing effects in the turbulence model comparative studies on supersonic steam ejectors. The novelty lies in the conclusions that consider the non-equilibrium condensing effects. From the systematic analysis and discussion above, the following key points have emerged from this study:

(1) The simulation results of the *k-ω* SST and *k-ε* Realizable agree with the experimental results in terms of the ejector performance. The simulation results of the *k-ω* Standard, *k-ω* SST, Linear RSM, and Transition SST are consistent with the experimental results in the shock wave type in the diffuser. The simulation results of the *k-ω* Standard, *k-ω* SST, Linear RSM, and Transition SST are consistent with the experimental results in the non-equilibrium condensation phenomenon. Therefore, the *k-ω* SST model is proposed to simulate supersonic steam ejectors;
(2) The number and type of shock waves predicted by various turbulence models are different. In the diffuser, the *k-ε* Realizable, the *k-ε* RNG, and the *k-ε* Standard predict a normal shock. The other four models predict a series of oblique shock waves;
(3) The Linear RSM, *k-ω* Standard, *k-ω* SST, Transition SST, and *k-ε* RNG predict the non-equilibrium condensation in the whole mixing chamber and diffuser while the *k-ε* Standard and the *k-ε* Realizable predict that only in the contraction of the mixing chamber;
(4) The *k-ω* Standard, *k-ω* SST, and Transition SST predict the boundary-layer separation phenomenon in the mixing chamber and diffuser, while the other models only predict that in the mixing chamber. In the mixing chamber of the ejector, two vortices are predicted by the above three models, while only one vortex is predicted by the other models.

## Figures and Tables

**Table 1:** Computation parameters.

*P*_m_ [Pa]	*T*_m_ [°C]	*P*_s_ [Pa]	*T*_s_ [°C]	*P*_d_ [Pa]
11906	51.3	1306	10.8	1350~1900

**Table 2:** Calculated results of the GCI method.

(*x*,*y*,*z*)/[mm]	*P*_1_/[Pa]	*P*_2_/[Pa]	*P*_3_/[Pa]	*e*_21_/(%)	*e*_32_/(%)	${GCI}_{fine}^{21}/(\%)$	${GCI}_{fine}^{32}/(\%)$
**(47, 0, 0)**	8014.17	6061.69	6030.98	24.36	0.51	1.96	0.16
**(85, 0, 0)**	1705.44	2302.26	2317.95	35.00	0.68	4.27	0.28
**(189, 0, 0)**	1425.12	1536.24	1547.09	7.80	0.71	3.11	0.07
**(261, 0, 0)**	1029.54	1026.48	1025.18	0.30	0.13	1.31	1.03

## Data Availability

The data presented in this study are available on request from the corresponding author.

## Nomenclature

*B*	[m^3^/kg]	Virial coefficients
*C*	[m^6^/kg^2^]	Virial coefficients
*CP*	[J/(kg∙K)]	Isobaric heat capacity
*E*	[J]	Total energy
*Er*	[–]	Entrainment ratio
*e*	[%]	Relative error
*F*	[N/m^3^]	Source term
*G*	[kg/s]	Mass flow rate
*h*	[J/kg]	Specific enthalpy
*hlv*	[J/(kg)]	Latent heat of condensation
*J*	[1/s]	Nucleation rate
*K*	[W/(m^2^∙K)]	Heat transfer coefficient
*k*	[m^2^/s^2^]	Turbulent kinetic energy
*kB*	[–]	Boltzmann constant
*Ma*	[–]	Mach number
*P*	[Pa]	Pressure
*R*	[–]	Gas-law constant
*r*	[m]	Droplet radius
*S*	[–]	Super-saturation ratio
*s*	[J/(kg∙mol∙K)]	Specific entropy
*ST*	[kg∙K/ (m^3^∙s)]	Viscous dissipative term
*T*	[K]	Temperature
*RSM*	[–]	Reynolds Stress Model
*RANS*	[–]	Reynolds Average Navier-Stokes
*N*-*S*	[–]	Navier-Stokes
*GCI*	[–]	Grid Convergence Index
**Special Characters**		
*β*	[–]	Liquid mass fraction
*Γ*	[kg/s]	Liquid mass generation rate
ρ	[kg/m^3^]	Density
γ	[–]	Specific heat capacities ratio
*μ*	[Pa/s]	Dynamic viscosity
*σ*	[N/m]	Liquid surface tension
*η*	[1/m^3^]	Droplet number density
*θ*	[–]	Non-isothermal correction factor
*v*	[m^2^/s]	Kinematic viscosity
*ε*	[m^2^/s^3^]	Turbulent dissipation rate
*τ*	[N/m^2^]	Stress tensor
*λ*	[W/(m∙K)]	Thermal conductivity
**Subscripts**		
*sat*	Saturation	
*m*	Motive steam	
*s*	Suction steam	
*d*	Discharge steam	
*l*	Liquid	
*v*	Vapor	
max	Maximum	
***	Critical	
-	Average	
*eff*	Effective	
*i*,*j*	Space components	
